# In vivo mapping of hemodynamic responses mediated by tubuloglomerular feedback in hypertensive kidneys

**DOI:** 10.1038/s41598-023-49327-3

**Published:** 2023-12-11

**Authors:** Blaire Lee, Dmitry D. Postnov, Charlotte M. Sørensen, Olga Sosnovtseva

**Affiliations:** 1 Department of Biomedicine, The University of Copenhagen, 2100 Copenhagen, Denmark; 2 CFIN Department of Clinical Medicine, Aarhus University, 1710 Aarhus, Denmark

**Keywords:** Chronic kidney disease, Characterization and analytical techniques

## Abstract

The kidney has a sophisticated vascular structure that performs the unique function of filtering blood and managing blood pressure. Tubuloglomerular feedback is an intra-nephron negative feedback mechanism stabilizing single-nephron blood flow, glomerular filtration rate, and tubular flow rate, which is exhibited as self-sustained oscillations in single-nephron blood flow. We report the application of multi-scale laser speckle imaging to monitor global blood flow changes across the kidney surface (low zoom) and local changes in individual microvessels (high zoom) in normotensive and spontaneously hypertensive rats in vivo. We reveal significant differences in the parameters of TGF-mediated hemodynamics and patterns of synchronization. Furthermore, systemic infusion of a glucagon-like-peptide-1 receptor agonist, a potential renoprotective agent, induces vasodilation in both groups but only alters the magnitude of the TGF in Sprague Dawleys, although the underlying mechanisms remain unclear.

## Introduction

Chronic kidney disease, mainly driven by diabetes and hypertension, affects about 10% of the global population and is defined by the functional and structural degradation of the kidney, impairing its ability to filter the blood. In investigating the influence of hypertension on the kidney, accumulating evidence suggests that microcirculatory hemodynamic changes occur in the pathogenesis of hypertension^1^.

Renal autoregulation is a crucial aspect of microcirculation that allows the kidney to maintain a stable renal blood flow and glomerular filtration rate despite the variations in blood pressure. Nephrons accomplish this by modulating the blood flow into the glomerular capillaries^2,3^. Two major mechanisms of renal autoregulation have been thoroughly investigated in connection to hypertension: myogenic response (MR) and tubuloglomerular feedback (TGF), which modulate the resistance of afferent arterioles periodically at $\approx$ 0.10 Hz and $\approx$ 0.033 Hz, respectively^4,5^. While the MR activates in response to increased afferent arteriolar transmural pressure, the TGF responds to the luminal Cl- concentration increase at the distal end of the thick ascending loop of Henle via the macula densa^6,7^. Notably, a comparison of the dynamic features of the TGF between normotensive Sprague Dawleys (SD) and spontaneously hypertensive rats (SHR) showed that while the prior had more stable and periodic TGF oscillations, the latter was found to be highly irregular^8–11^. The traditional dogma that views the TGF as a single nephron event has been shifting: studies have shown that nephrons can form synchronized nephrovascular ensembles via TGF signal propagation along the common artery’s arteriolar branches^12–17^. This urges an investigation into nephron ensembles of SDs and SHRs.

An imbalance in the RAAS system and upregulated SGLT2 activity are major drivers of glomerular hyperfiltration and tubular hyper-reabsorption in the progression of CKD in diabetes^18^. Conventional strategies such as angiotensin-converting-enzyme (ACE) inhibitors and angiotensin-II-receptor blockers (ARBs) are renoprotective and reduce the risk of end-stage renal disease (ERSD)^19^. In treating diabetes, exenatide, a GLP-1 receptor agonist (GLP-1RA), is a promising pharmacological agent for reducing hyperglycemia and blood pressure, but it has also shown signs of renoprotection^20^. The decline in renal function threatens diabetic patients, with symptoms manifesting in the form of hypertension, sodium retention, albuminuria, glomerular hyperfiltration, and microvascular damage, to name a few^21^. In human and animal models, studies have shown that GLP-1RAs mitigate some of these risk factors through renal vasodilation, diuresis, natriuresis, and blood pressure reduction^22–25^. In Wistar Kyoto rats, acute exenatide infusion induced vasodilation in the kidney and increased the glomerular filtration rate (GFR)^26^. Moreover, infusing GLP-1 in rats caused natriuresis that is linked to the inhibition of tubular sodium reabsorption, which could potentially enhance the TGF-mediated vascular response via increased sodium chloride delivery to the macula densa^27^. Although these studies allude to how GLP-1RAs might affect renal hemodynamics, the real-time effects of GLP1-RA on the TGF-induced hemodynamic changes in vivo remain largely unexplored.

Compared to SDs, the SHRs have reduced GLP-1R expression in the pre-glomerular vasculature, where TGF and MR modulations occur^28^. Yet, Ronn et al. demonstrated that the intrarenal infusion of GLP-1 increased the renal blood flow and urinary flow rate in SHRs^29^. Savignano et al. observed diminished diuresis and natriuresis with no renal hemodynamic changes in response to systemic GLP-1 administration in SHRs compared to SDs^30^. These studies indicate that the TGF dynamic in response to GLP-1RA may differ between the SDs and SHRs. However, a study under the same experimental condition and mode of administration is required to compare the effect of GLP-1RA on the TGF-induced hemodynamics between SDs and SHR. Unfortunately, experimental approaches for evaluating TGF-mediated oscillations in vivo remain limited. Micropuncture, albeit the current gold standard, is only suitable for measuring [NaCl], single nephron GFR, and tubular flow and pressure changes elicited by the TGF in three nephrons maximally. Particularly for the TGF system, which is highly sensitive to physiological and experimental variations, a prolonged, non-contact recording method is optimal.

Laser speckle contrast imaging (LSCI) is a robust, minimally invasive system for monitoring surface blood flow in various tissues, such as the skin, brain, and kidney, in real-time in vivo^15,31,32^. The LSCI technique can assess blood flow in microvessels (arising from the efferent arterioles) or the whole kidney perfusion, depending on the magnification: this is critical when the hemodynamic across the kidney surface is heterogeneous^13^. Such a system has been employed in the past to investigate the effect of drug distribution on the renal surface^33,34^ and to demonstrate that nitric oxide affects the TGF and myogenic-driven hemodynamics^16^. Recently, Postnov et al. introduced high-resolution laser speckle imaging of renal blood flow. They estimated the frequency and phase differences in rat kidney microcirculation: the angiotensin II infusion promoted stronger synchronization while acetylcholine infusion induced complete desynchronization^12^. Previously, we proposed a multi-scale LSCI (msLSCI) system suitable for evaluating tissue circulation at two different imaging scales simultaneously: high-zoom (small field-of-view) and low-zoom (large field-of-view)^35^. Using the high-zoom component of the system, we demonstrated the TGF-induced hemodynamic changes in a population of microvessels associated with nephrons during systemic infusions of a Na-K-2Cl inhibitor (furosemide) and a sodium-glucose co-transporter inhibitor (phlorizin) in Sprague Dawleys^36^.

In this study, we employed the multi-scale LSCI to simultaneously assess the renal microcirculation over the kidney surface and in individual micro-vessels of SDs and SHRs. By applying the time-frequency super-resolution (SLT) analysis^37^, we aimed to discern the dynamical features of TGF-mediated vascular responses and synchronization patterns in the two rat strains. Furthermore, we evaluated the TGF-induced hemodynamic responses in vivo to the systemic administration of GLP-1R agonist (exenatide) in the presence (SDs) and low expression (SHRs) of GLP-1Rs in the preglomerular vascular smooth muscle cells.

## Results

### Hemodynamic responses between SD and SHR

Animals were weighed prior to the experiment. The average weights were 344$\pm 15$ g and 251$\pm 16$ g for the SD and SHR groups, respectively. Arterial blood pressure was monitored during the saline infusion (control period). The SHRs had a substantially higher blood pressure at 140$\pm 9$ mmHg compared to the SDs (122$\pm 9$ mmHg).

**Figure 1:**
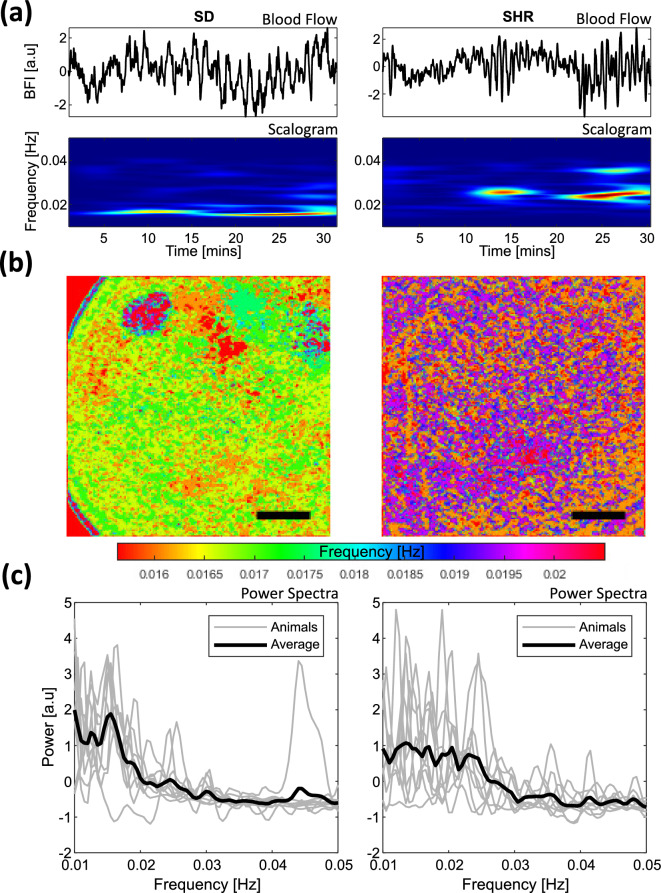
Representative low zoom recordings. TGF signals can be detected in the renal hemodynamics of SDs and SHRs. (**a**) Exemplary normalized blood flow time series and time-frequency scalogram show TGF-mediated oscillations in an SD (left) and SHR (right). (**b**) The dominant TGF frequency maps in the SD (left) and SHR (right) kidneys show spatial heterogeneity across the renal surface. Scale bar $\approx 1$ mm. (**c**) Power spectra spatially averaged over the field of view showing a single dominant TGF frequency peak in SDs (left) and several peaks in SHRs (right). The gray plots represent the power spectra of individual animals, and the black plot represents a spectrum averaged across the animals.

Across all animals in SDs and SHR groups, we observed TGF-mediated changes in the blood flow dynamics across the kidney surface with the low zoom settings of the msLSCI. Scalograms obtained with SLT analysis of the blood flow clearly distinguished the TGF-mediated changes within the TGF frequency range (0.015–0.04 Hz) in both SDs and SHRs (Fig. 1a). To demonstrate the heterogeneity across the renal surface, we computed the power spectrum of the time series extracted from every pixel, mapped the most prominent frequency peaks, and applied a sliding window median filter of 20x20 pixels to reveal the spatial frequency patterns. We found spatial frequency heterogeneity in both groups, although the ranges of dominant frequencies differ (Fig. 1b). To verify the existence of TGF-induced renal hemodynamics across the kidney surface, low zoom blood flow signal was analyzed in the time-frequency domain via SLT analysis. Every animal in each group (SD: n = 9) and (SHR: n = 9) exhibited a signal within the TGF frequency range at the kidney surface, indicating that the TGF was activated. A summary of the hemodynamics represented as time-averaged power spectra for each animal is demonstrated in Fig. 1c. When individual power spectra were averaged to a single spectrum for each group, the SD group exhibited a single dominant TGF peak in the power spectrum. In contrast, the SHR group showed several less prominent peaks.

**Figure 2:**
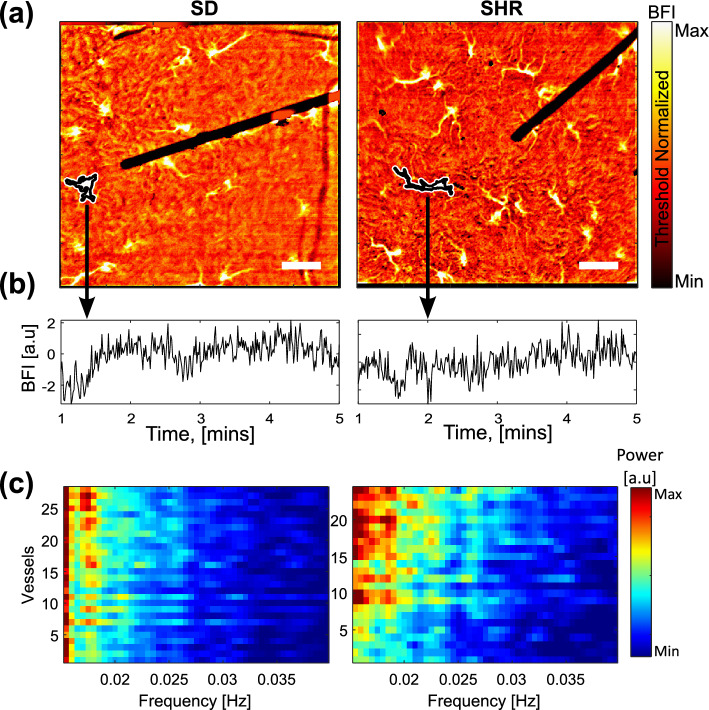
High zoom recordings. (**a**) Time-averaged blood flow maps show similar microvasculatures in SDs (left) and SHRs (right). The individual vessels can be segmented (black boundaries enclosing the vessels). The color threshold is normalized to exclude the myograph wire. Scale bar $\approx 200\,\upmu$ m. (**b**) Blood flow time series can be extracted from the segmented region for further analysis. (**c**) Representative example of frequency distributions within TGF frequency range over segmented vessels in SDs (left) and SHRs (right). The color scale encodes the power of the identified spectral peaks.

We evaluated the hemodynamic features in individual microvessels that can be visualized on the renal surface with high zoom settings of the msLSCI. The visualized vessels are located downstream from the efferent arterioles and positioned just underneath the renal capsule. Occasionally, they are called “star vessels” for their morphological resemblance to a star^38–40^. Similar vessel morphology was observed in SDs and SHRs in the time-averaged blood flow map (Fig. 2a), which enabled the application of the same vessel segmentation process. From the segmented vessels, blood flow variations were extracted as shown in Fig. 2b. Spectral analysis of the segmented vessels revealed that the SHRs exhibit more spectral peaks in the TGF frequency range than the SDs, in agreement with the low zoom analysis (Fig. 2c).

**Figure 3:**
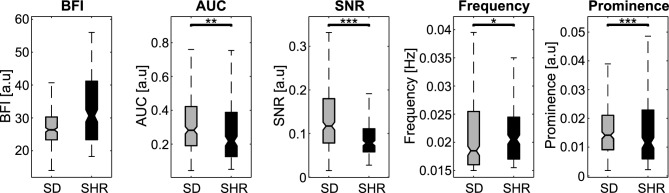
Parameters of TGF-mediated responses between SDs and SHRs in control. SHRs show a tendency toward a higher BFI than the SDs. Parameters associated with TGF-mediated hemodynamics, i.e., AUC, SNR, and prominence, are consistently lower in SHRs. Dominant TGF frequency is higher in SHRs. The center line of the box plot indicates the median, and the bottom and the top edges represent the $25{\textrm{th}}$ and the $75{\textrm{th}}$ percentiles of the data, respectively. Significant star scheme: * represents $P<0.01$, ** represents $P<0.001$, and *** represents $P<0.0001$.

We analyzed the blood flow time series of every segmented microvessel in the high-zoom data. Several TGF-associated hemodynamic features were quantified from the blood flow signals. The differences between the SDs and SHR groups were evaluated with the Mann-Whitney U test (Fig. 3). The time-averaged blood flow index (BFI), which is proportional to the linear flow velocity, was similar between the two strains. The overall magnitude of the TGF-mediated oscillation (AUC) was significantly higher in SDs ($P<0.001$). In agreement with the AUC, the signal-to-noise ratio (SNR) of the TGF signal compared to the noise level was higher in SDs ($P<0.0001$). SHRs exhibited faster dominant TGF frequencies than the SD group ($P<0.01$), but the prominences of those peaks were lower ($P<0.0001$). Note that the BFI, AUC, and SNR characterize the overall hemodynamics, while frequency and prominence describe single peak characteristics in the power spectrum.

**Figure 4:**
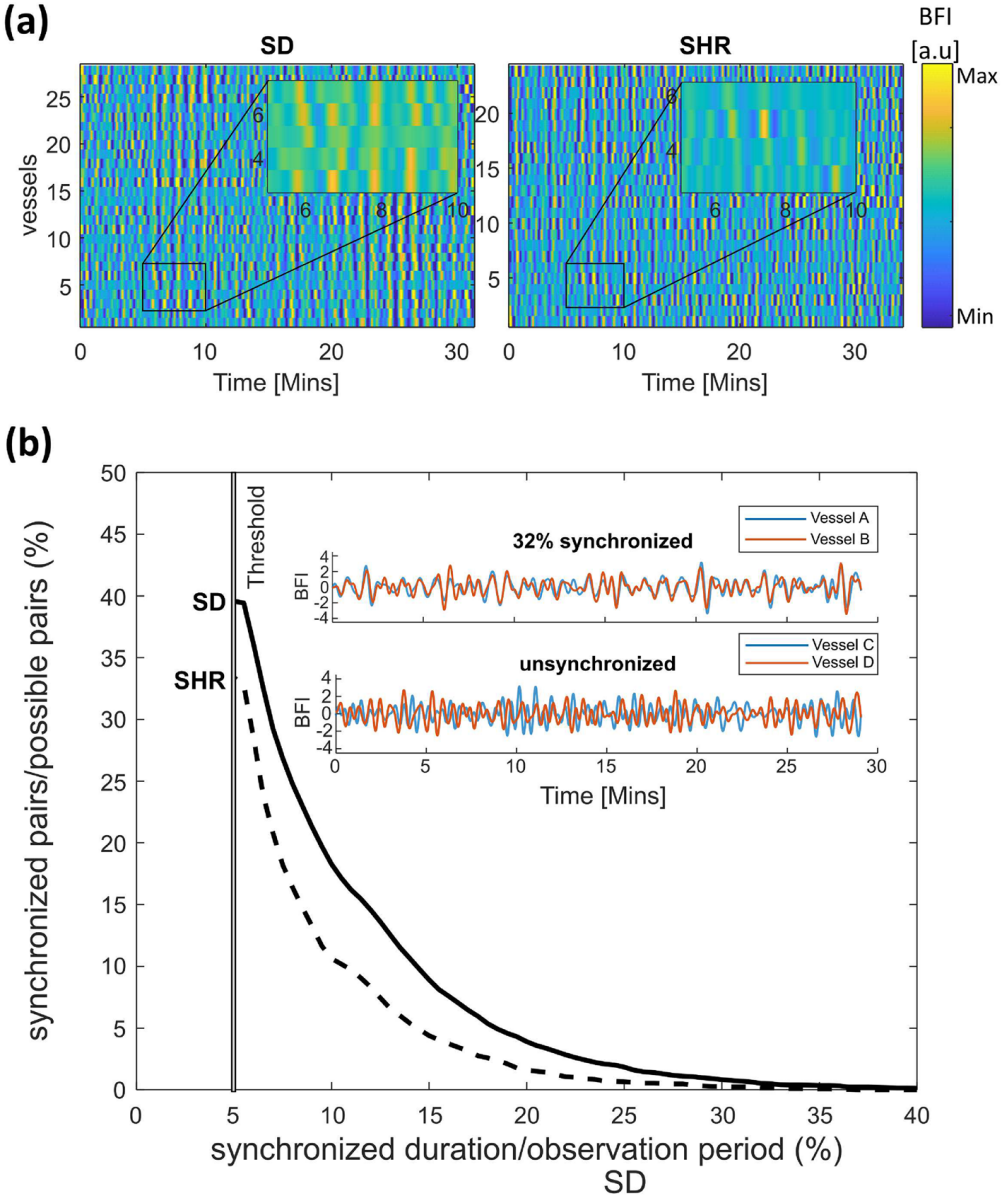
Synchronous patterns. (**a**) Representative renal hemodynamics in individual SDs and SHRs. Hemodynamic variations in over 20 vessels can be visualized as carpet plots of the filtered blood flow for SD (left) and SHR (right). Insets show TGF oscillations with phase and amplitude variations. (**b**) Synchronized pairs as a function frequency-locked duration over all animals. SHRs (dashed) show reduced synchronization compared to SDs (solid). Inset: the blood flow traces shows pairs of vessels that are 32% (top) and 0% (bottom) synchronized out of the entire observation time in a normotensive rat.

The high zoom component of the imaging modality enabled investigation into how TGF-mediated changes in one vessel can affect hemodynamics in neighboring microvessels. Fig. 4a demonstrates the collective oscillation mediated by the TGF in individual microvessels: the striated patterns illustrate periods of synchronized behavior among vessels. In both SDs and SHRs, there were clusters of vessels that exhibited cooperative TGF-induced changes (insets in Fig. 4a). We assess synchronization between all possible pairs of vessels (SD: n = 5725; SHR: n = 2947) by setting synchronization conditions: TFG-mediated oscillations were considered synchronized when their instantaneous frequencies were locked for a minimum of two periods. Figure 4b demonstrates that the SHRs have fewer synchronized vessel pairs at any given synchronization duration. Although we implement a minimum locking time of about two periods for a pair of vessels to be considered synchronized, the difference can be observed without such a limitation.

### Renal hemodynamics under the administration of GLP-1R agonist

Arterial blood pressure was monitored during the saline infusion (control) and the intervention period. GLP-1RA induced no change in the blood pressure in the SD group from 122$\pm 9$ mmHg to 119$\pm 14$ mmHg. In the SHR group, paired t-test revealed a substantial decrease in BP from 140$\pm 9$ mmHg to 126$\pm 13$ mmHg ($P<0.01$).

**Figure 5:**
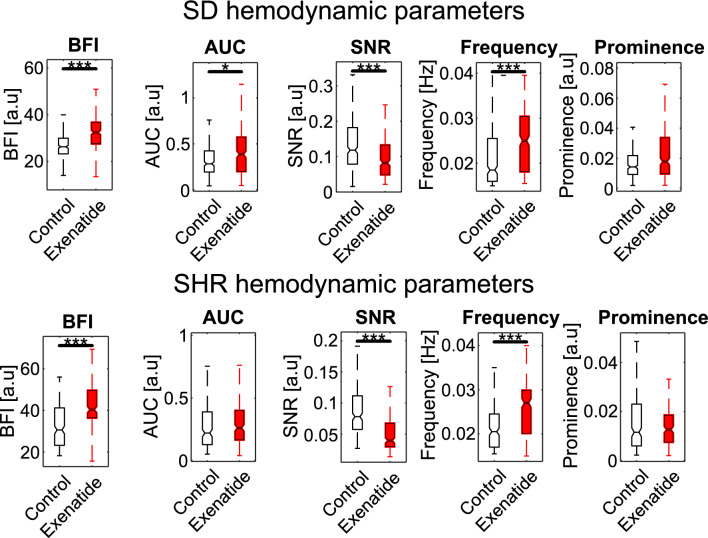
Hemodynamic parameters show similar responses to GLP-1R agonist (exenatide) intervention in SDs and SHRs. Both groups show increased microcirculatory blood flow (BFI). The AUC is larger in SDs but not in SHRs. SNR of the TGF signal is lower after infusion in both groups. The dominant TGF frequency is higher, but the prominence remains the same in both groups. The center line of the box plot indicates the median, and the bottom and the top edges represent the $25{\textrm{th}}$ and the $75{\textrm{th}}$ percentiles of the data, respectively. Significant star scheme: * represents $P<0.01$, and *** represents $P<0.0001$.

After evaluating the differences between SDs and SHRs during baseline measurement, we assessed how the exenatide could perturb renal microcirculatory hemodynamics. Exenatide infusion increased the BFI (SD: $P<0.0001$; SHR: $P<0.0001$) in both groups. Notably, the magnitude of the TGF-mediated changes (AUC) changed significantly only in the SD group ($P<0.001$). The SNR decreased (SD: $P<0.0001$; SHR: $P<0.0001$) in both groups, and the dominant TGF is shifted to higher frequencies (SD: $P<0.0001$; SHR: $P<0.0001$), but their prominences did not change. Plots for hemodynamic parameters are shown in Fig. 5. A summary of the statistics can be found in Table S1.

## Discussion

### Summary of the main perspectives

Altered renal microcirculation is linked to impaired renal function in diabetes and hypertension. Micropuncture technique for measuring single nephron tubular flow changes can only probe two to three nephrons at a time, which is insufficient in number– a rat kidney has more than 30,000 nephrons^41^. First, this study employs msLSCI to discern the TGF-mediated microcirculation in ensembles of nephrons in SDs and SHRs in vivo. Second, we expand on the current knowledge about nephron-nephron synchronization in normotensive and spontaneously hypertensive rats. Third, we demonstrate how acute GLP-1R activation can alter the renal hemodynamics in SDs and SHRs, which may partially explain its role in renoprotection.

At baseline (saline administration), the SDs and SHRs exhibit different TGF-mediated oscillatory hemodynamics, measured by our parameters. The blood flow oscillation driven by the TGF mechanism in Sprague-Dawleys, and the irregular oscillations in SHRs were first observed decades ago in single nephrons with the micropuncture technique^8,42,43^. Another study made a similar observation in animal models with renovascular hypertension or acute hypertension induced by renal artery clipping^44^. It has been suggested that the irregular oscillations observed in SHRs may indicate impaired autoregulation. In alignment with previous studies, our results indicate that TGF-mediated oscillation is weaker (AUC) and noisier (SNR) in SHR compared to SD (Fig. 3). We can speculate that the weaker TGF magnitude could be due to the reduced capacity of the TGF response at higher systemic blood pressure. Interestingly, the SHRs had a higher dominant TGF frequency than the SDs, which may be due to higher flow. However, these parameters are single peak metrics of the power spectrum, and further investigation is required to explain the difference.

The conventional dogma is that nephrons are independently responsible for regulating their own blood flow, and the sum of their activity results in whole kidney autoregulation. However, the kidney possesses a sophisticated arterial architecture that manages to maintain a relatively stable blood flow. We know now that 50 % of the afferent arterioles are arranged in pairs and triplets that are supplied by the same cortical radial artery^45^, enabling vascular communication via TGF-mediated signals^46^, which potentially contributes to efficient autoregulation^47^. Marsh et al.^48^ demonstrated such interactions in a 16-nephron vascular network model and predicted synchronous patterns in paired nephrons. Along with technical advances, the modern paradigm has been nudging for ways to assess interactions among ensembles of nephron-vascular network^17,49^. Fortunately, the development of laser speckle flowmetry imaging has enabled observation of TGF-conducted nephron synchronization over the whole renal surface in identified microvessels^15^. With the technique, it was found that synchronized nephron ensembles exist^13^, and they could be altered with systemic and intra-renal drug administrations^12,16^. In hypertensive animal models, the TGF-initiated nephron-nephron interaction is altered^11,50^. Specifically, Sosnovtseva et al. found that the synchronous dynamic between the TGF and myogenic (MR) mechanisms among neighboring nephrons (sharing the same cortical radial artery) is reduced in hypertensive rats^14^. 11 pairs and nine pairs of nephrons were measured with a servo-nulling technique in SHRs and normotensive rats, respectively, revealing important differences in nephron synchronization between kidneys of normotensive and hypertensive conditions. However, the small sample size of nephrons is a significant limitation of these previous studies. To validate the difference in synchronization patterns between the two rat strains in a larger set of nephrons, we analyzed about 30 microvessels (associated individual nephrons cortical^15,38^) per animal, resulting in the assessment of 5725 (SD group) and 2947 (SHR group) non-repeating pairwise combinations of vessels. Our results found that 20 % of the possible pairs were synchronized in the SD group, while only 10 % of the possible pairs were synchronized in SHRs for 10 % of the observation period. For context, a pair of vessels that are synchronized for a mere 32% of the observation period (30 min) still exhibits a high degree of signal synchronization (inset of Fig. 4b). However, since this study does not validate the anatomical pairing of nephrons, the synchronization can only be quantified as an observed probability. Initially, Marsh et al.^51^ confirmed the existence of electronic signal conduction along the preglomerular vasculature that allows for synchronization. In adjoining nephrons, the magnitude of vascular response of one arteriole becomes weaker as the distance from the stimulation site increases^52^. Furthermore, Chen et al. showed that the TGF-mediated nephron-nephron interaction is more robust in SHRs, and the interaction strength depends on the distance separating the glomeruli^50^. In this study, we observed a similar trend by directly assessing the cross-correlation of TGF-mediated oscillations between nephron pairs: a decline in correlation degree as the distance increases between two vessels (Fig. Plus, the SHR group exhibited a stronger negative correlation than the SDs, but the functional justification for this phenomenon in hypertensive rat kidneys remains unanswered. Perhaps the TGF-mediated collaboration among nephron ensembles aids in managing the persistently elevated systemic pressure.

Although GLP-1RA is primarily used as a treatment for type 2 diabetes, clinical studies have shown that it can also be renoprotective^53^. But why renal GLP-1R activation leads to renoprotective effects is not fully understood. This study highlights the hemodynamic changes that may be associated with the renoprotective effect of GLP-1RA through the TGF system. In normotensive rats, Fig. 5 shows that exenatide can induce a potent increase in the microcirculatory blood flow (BFI) and enhance the magnitude of the TGF-mediated hemodynamic (AUC) at stable blood pressure. A plausible explanation for the enhanced TGF magnitude is that the increased flow leads to greater sodium chloride delivery to the macula densa (via reduced NHE3-dependent tubular sodium reabsorption^54^), activating the TGF system^55^, which can be renoprotective. In hypertensive rats, GLP-1RA did not have an effect on the TGF magnitude, even though there was a substantial increase in the BFI and a decrease in blood pressure. The unchanged AUC in SHRs could indicate reduced autoregulatory capacity when the kidney is faced with acute systemic pressure changes. Possibly, the TGF-mediated hemodynamic change did not occur in the SHR group due to reduced GLP-1R expression in the smooth muscle cells of preglomerular vasculature, as supported by Jensen et al.^28^. However, other pathways that GLP-1RAs could affect renal hemodynamics should be considered in interpreting our results. For example, clinical evidence suggests that GLP-1RA might counteract angiotensin II activity (RAAS)^56,57^, which is a sign of renoprotection against diabetic nephropathy^58^. Plus, certain neural and metabolic pathways are also thought to be involved in GLP-1RA-induced renal hemodynamic changes^59^. Further investigation is needed to discern their independent contribution to renoprotection. The efficacy of GLP-1RA on the kidney may also differ based on the mode of delivery^60^, the duration of treatment, and the severity of renal function impairment^23^. A future study with longer treatment durations and measurements at various stages of chronic kidney disease progression could address some of the variability mentioned above. In a mouse kidney model of diabetic nephropathy, GLP-1Rs were found in the glomerular capillaries and vascular walls but not in the tubules^61^. It would be interesting to investigate how acute GLP-1R activation affects the renal hemodynamics in these mice, furthering our understanding of how GLP-1RA may aid in slowing down the functional degradation in diabetic nephropathy. Finally, we acknowledge that there is an inherent limitation of our imaging system. Since we only access the cortical surface of the kidney, the results presented here lack insights into the deeper cortical and medullary hemodynamics.

To summarize, the microvascular network of the kidney is sophisticated in function and structure. This study demonstrates that systemic hypertension adds another layer of complexity to the hemodynamics mediated by the TGF, not just in terms of individual nephrons but also as synchronized nephron ensembles. Furthermore, the present study unveils some in vivo functional relevance of GLP-1R in the preglomerular vasculature: the enhancement of TGF-mediated hemodynamic oscillation in normotensive rats but not in hypertensive rats. Finally, our data suggest that GLP-1-induced renoprotection does not arise from TGF-mediated microcirculatory changes in hypertensive rats. Nevertheless, many questions remain as to the efficacy of GLP-1RA in renoprotection, and this study is just one piece of the puzzle.

## Methods

### Surgical procedure

The rats were anesthetized with a 5 % sevoflurane gas mixture in an induction box. After the toe pinch test, the animals were laid ventral side up and maintained under anesthesia at 1.8 % through a gas mask. The body temperature on a surgical heating table was maintained at 37$^{\circ }$C. A small horizontal incision was made across the thoracic region to isolate the left jugular vein, the trachea, and the right carotid artery. The left jugular vein was cannulated with a polyethylene tube (PP25, Smith Medicals) for constant saline or Exenatide (CAS 141758-74-9, Santa Cruz Biotech) infusion at 50 $\upmu$l per minute. After, the right carotid artery was cannulated with a larger polyethylene tube (PE50, Smith Medicals) to monitor the arterial blood pressure. A tracheotomy was performed for artificial ventilation with a volume-controlled respirator (7025, Ugo Basile) and anesthesia delivery (60 strokes/min). A Laparotomy was performed to expose the left kidney and stabilized with an agarose solution (0.5 %) to maintain hydration in a 3D-printed kidney-shaped frame. A glass coverslip was laid over the kidney, and a thin myograph wire bent at an acute angle was placed inside the imaged field of view. The left ureter was cannulated (PP10) for sample collection and free flow of the urine.

### Exenatide preparation

The drug was obtained from Santa Cruz Biotechnology in a powder form with a 97 % purity. A 50 $\upmu$M stock solution was made with a 1 % BSA solution, aliquoted 10 $\upmu$L in Eppendorf tubes, and stored at -20$^{\circ }$C. The tubes were thawed and diluted to a final concentration of 0.1728 $\upmu$M solution with a 1 % BSA for each experiment^29,62^.

### Experimental timeline

**Figure 6:**
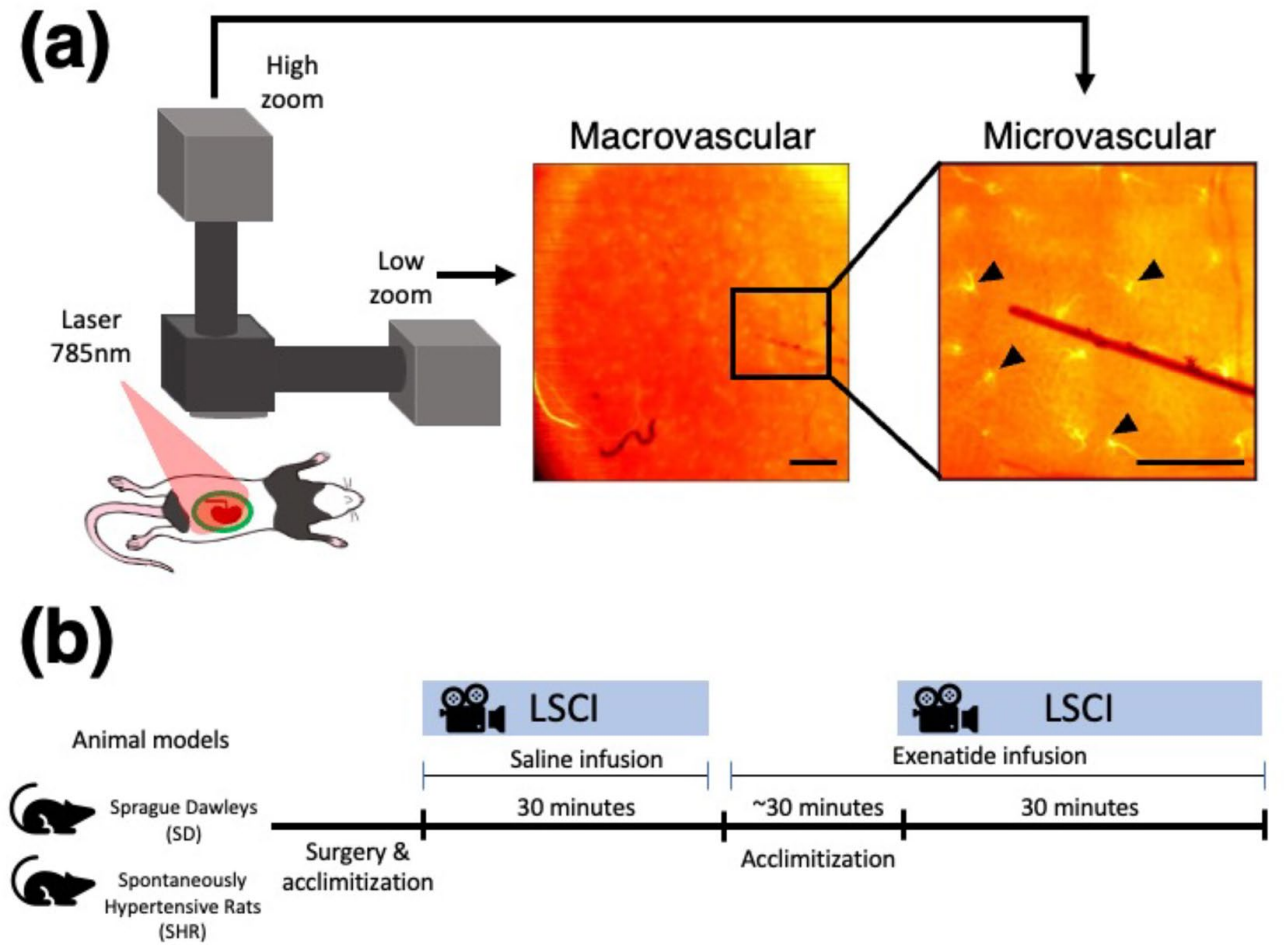
In vivo blood flow imaging setup and experimental scheme for systemic exenatide administration in rats. (**a**) High-zoom and low-zoom cameras can simultaneously record renal surface perfusion at different scale resolutions. The black arrows point to microvessels that arise from efferent arterioles. A sufficient acclimation period is given to the animals until stable blood pressure can be maintained. Scale bar macro $\approx 1$ mm; micro $\approx \, 200\,\upmu$m.

Experiments alternated between SDs and SHRs each day to minimize surgical bias. Each experiment began with a control imaging period of 30 min. Then, the exenatide infusion was initiated at 50 $\upmu$L, and following an acclimation period of 30+ min, an intervention recording period of another 30 min took place. During the control period, a 1 % BSA saline solution was systemically infused through the jugular vein catheter at 50 $\upmu$L. At the end of the control period, the saline was replaced with the exenatide solution and infused continuously until the end of the experiment. Raw speckle images were captured during the saline infusion and the intervention period. Refer to Fig. 6 for the illustration of the experiment.

### Blood flow imaging set up

The construction of the multi-scale laser speckle contrast imaging (msLSCI) and the in-depth data analysis can be found in^35^. One video zoom lens was set to 4.5 X magnification (1.2$\upmu$m/pixel size) to record the microcirculation, and the second was set to 1 X magnification (5.5$\upmu$m pixel size) to record the whole kidney blood flow. CMOS sensor cameras were connected to the video zoom lenses (acA2040-90umNIR, Basler). Volume Holographic Grating stabilized diode (LP7850-SAV50, 785 nm Thorlabs) was used to illuminate the surface of the renal tissue. The frame rate was set to 50 Hz and an exposure time of 5ms. Recordings took place for 30 min during the control period and after the initiation of exenatide infusion, resulting in 180,000 image frames per experiment for each magnification arm (high zoom and low zoom data).

### Data processing and signal analysis

All data were analyzed offline using MATLAB (MathWorks). Before the speckle contrast analysis, the high-zoom raw data underwent an image registration step for motion correction, using the myograph wire as a reference mark. The low zoom data did not require a motion correction because the movements were sub-pixel, i.e., the range of translational movement in the x-y plane was within 5.5 $\upmu$m. Next, we computed the temporal speckle contrast *K* as the ratio of the standard deviation to the mean pixel intensity for a temporal kernel of 25. Finally, we calculated the blood flow index (BFI) using $BFI = 1/K^2$.

Low zoom data analysis: first, a binary mask was created with an intensity threshold to exclude pixels covering non-tissue areas such as the kidney frame, fat tissue, and the myograph wire in the blood flow data. Then a Gaussian filter was applied to every frame and spatially averaged to produce a single blood flow time series representing the global renal surface blood flow. To produce the scalogram and the power spectra shown in Fig. 1 a and c, and to verify the presence of TGF frequencies in each animal, the time-frequency super-resolution spectral estimation (SLT) analysis was applied^37^. SLT was selected over continuous wavelet transform due to its superior performance in localizing transient oscillations in both time and frequency of biological data. The frequency of interest was set from 0.01 to 0.05 Hz at 80 bins, with wavelets spanning 15 cycles and the minimum and maximum order set to 1 and 50, respectively. To produce a spatial frequency map in Fig. 1b, discrete Fourier transform was applied to $1200\times 1200\times 180{,}000$ matrix data along the time (third) dimension. In this case, FFT was chosen over SLT for computational efficiency. Then a sliding window median filter with a neighborhood size of $20\times 20$ pixels was applied.

High zoom data analysis: individual microvessels (star vessels) were segmented, and blood flow time series were extracted from each vessel. 322 and 227 vessels were segmented for the hemodynamic analysis in SDs and SHRs, respectively. To produce time-averaged power spectra for each vessel, the SLT analysis was applied to individual vessel time series with the same settings as the low zoom analysis. After, several TGF signal parameters were extracted to analyze the TGF-driven hemodynamic changes:

- BFI was calculated by temporally averaging the blood flow time series over the observation period;
- The AUC was computed by approximating the integral of the power spectrum of the filtered signal via the trapezoidal method between the frequency of interest (0.015–0.04 Hz);
- The signal-to-noise ratio (SNR) was calculated by taking the ratio between the signal’s power and the noise’s power.
- Frequency of the most prominent (dominant) peak in the range of 0.015–0.04 Hz;
- Prominence measures how much the peak stands out due to its intrinsic height and its location relative to other peaks detected within 0.015–0.04 Hz;

### Synchronization analysis

All possible pairs were compared to analyze the synchronization among observed vessels in each animal. 5725 and 2947 vessel pairs were analyzed in SDs and SHR groups, respectively. For vessel pair A and B, instantaneous frequencies were computed and parsed for consecutive durations of matching dominant frequencies, referred to as “synchronized duration” henceforth. Two criteria were implemented for calculating the synchronized duration: (1) two synchronized durations interrupted by less than the shortest possible period (25 seconds) were concatenated into a single duration, and (2) any synchronized duration of fewer than two periods was disregarded. To compare the synchronization between SDs and SHRs, the synchronized duration was plotted as a percentage of the observation time of each experiment, and the number of synchronized pairs was plotted as a percentage of all possible pairs. Figure 4 was produced through this analysis.

### Correlation analysis

All possible pairs were identified to measure the similarity among observed vessel pairs in each animal, and cross-correlations of respective pairs of filtered signals were computed. Their Euclidean distances, in pixels, were also calculated. Finally, the signal similarity was plotted as a function of distance in Fig. S1, and linear regression fits were performed using the linear least squares method. The process was repeated for each experimental condition.

### Statistical analysis

Physiological measurements (SD: n = 9; SHR: n = 9) were compared using paired t-test (two-tailed). Distributions of TGF parameters were tested for normality using the Anderson-Darling test, and all rejected the null hypothesis of normality at the default 5% significance level. Non-parametric tests were used for subsequent comparisons of non-normally distributed data. The parameters between SD (n = 322) and SHR (n = 227) groups were compared using the two-sided Mann–Whitney U test (Fig. 3) at a 5% significance level. In addition, a two-sided Wilcoxon signed-rank test was used to compare the parameters (paired data) between the saline and the exenatide infusion period for both the SD (n = 322) and the SHR (n = 227) groups (Fig. 5) at a 5% significance level. Refer to Table S1 for the statistics table. Data are presented as box and whisker plots with the central mark for the median value and the top and the bottom edges indicating the 25th and the 75th percentiles. The whiskers extend to extreme data points that are not outliers. P values below 0.01 were considered significant. The statistics table can be found in the supplementary material.

### Ethical approval

The Danish National Animal Experiments Inspectorate approved all procedures (no.2020-15-0201-00547). All methods were performed in accordance with the relevant EU guidelines and regulations. The study is reported in accordance with ARRIVE guidelines. The animals received a 7-day acclimatization period and were caged in a 12-hour light cycle facility with ad libitum access to food and water. Male Sprague Dawleys (n = 9, RjHan: SD) and spontaneously hypertensive rats (n = 9, SHR/KyoRj) were used for this study (Janvier, France).

### Supplementary Information

Supplementary Information.

## Data Availability

The datasets generated during and/or analyzed during the current study are available from the corresponding author upon reasonable request.
