# Supplementary material for: In vivo mapping of hemodynamic responses mediated by tubuloglomerular feedback in hypertensive kidneys
Source: Sci Rep. 2023 Dec 11;13:21954. doi: 10.1038/s41598-023-49327-3 (PMC10713540; doi:10.1038/s41598-023-49327-3)
Supplement: Supplementary file 1 — Supplementary Information. [file 41598_2023_49327_MOESM1_ESM.pdf]

SD (Control)

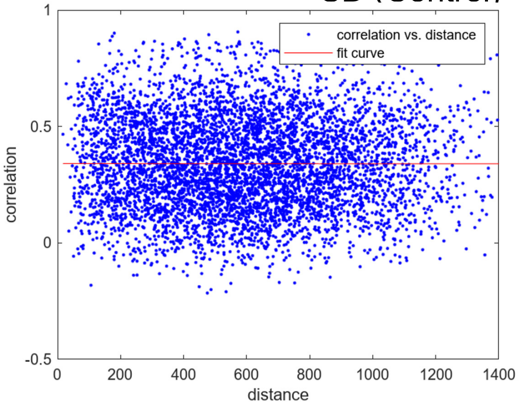


```
fitresult =
     Linear model Poly1:
     fitresult(x) = p1*x + p2
     Coefficients (with 95% confidence bounds):
       p1 =  -7.082e-07  (-1.856e-05, 1.714e-05)
       p2 =      0.3407  (0.3289, 0.3524)
gof =
           sse: 229.3687
       rsquare: 1.0576e-06
           dfe: 5723
    adjrsquare: -1.7368e-04
          rmse: 0.2002
```

SD (Exenatide)

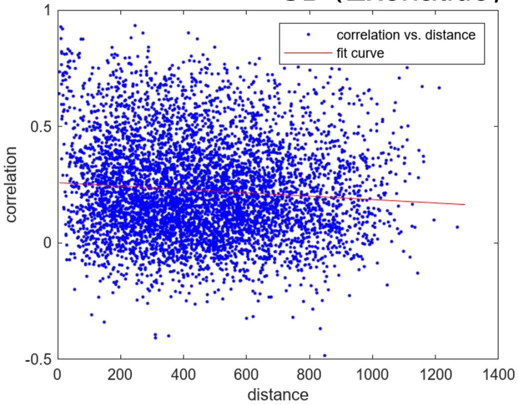


```
fitresult =
     Linear model Poly1:
     fitresult(x) = p1*x + p2
     Coefficients (with 95% confidence bounds):
       p1 =  -7.291e-05  (-9.549e-05, -5.033e-05)
       p2 =      0.2591  (0.2474, 0.2708)
gof =
           sse: 251.5443
       rsquare: 0.0070
           dfe: 5723
    adjrsquare: 0.0068
          rmse: 0.2097
```

SHR (Control)

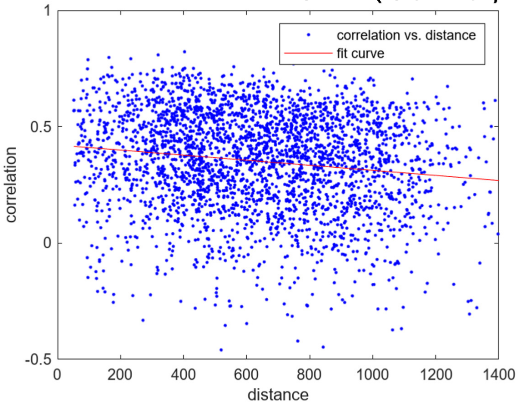


```
fitresult =
     Linear model Poly1:
     fitresult(x) = p1*x + p2
     Coefficients (with 95% confidence bounds):
       p1 =  -0.0001089  (-0.0001353, -8.254e-05)
       p2 =      0.4213  (0.4029, 0.4398)
gof =
           sse: 132.2258
       rsquare: 0.0217
           dfe: 2945
    adjrsquare: 0.0214
          rmse: 0.2119
```

SHR (Exenatide)

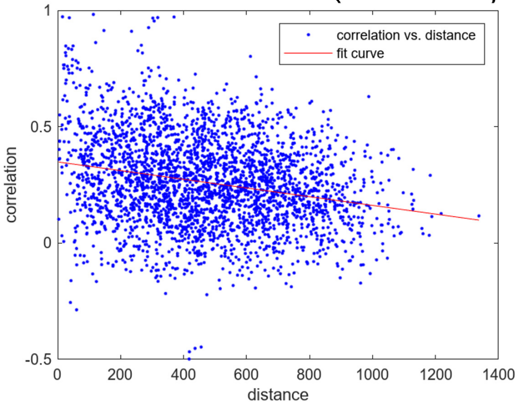


```
fitresult =
     Linear model Poly1:
     fitresult(x) = p1*x + p2
     Coefficients (with 95% confidence bounds):
       p1 =   -0.000187  (-0.0002156, -0.0001585)
       p2 =      0.3488  (0.3336, 0.3639)
gof =
           sse: 108.3483
       rsquare: 0.0531
           dfe: 2945
    adjrsquare: 0.0528
          rmse: 0.1918
```

Figure 1. Linear regression shows the relationship between signal correlation and the distance increases among vessels under various conditions. There is a faster decline in correlation as the distance increases in SHRs compared to SDs during control (left column). The slope becomes two-fold steeper during the exenatide infusion in both groups (right column). Blue dots represent every possible non-repeating vessel pair.

	SD Control (n=322)			SHR Control (n=227)			Mann-Whitney U		
Parameters	Median	Quantiles (25/75)		Median	Quantiles (25/75)		pVal	zVal	ranksum
BFI	26.3603	23.2859	30.3307	30.6420	23.3091	41.2179	2.11E-02	4.0312	95929
AUC	0.2820	0.1896	0.4229	0.2178	0.1254	0.3884	1.87E-04	3.7362	95389
SNR	0.1171	0.0778	0.1803	0.0780	0.0575	0.1113	8.47E-14	7.4628	102210
Frequency	0.0185	0.0160	0.0255	0.0205	0.0170	0.0245	2.63E-03	-3.0085	83051
Prominence	0.0141	0.0090	0.0211	0.0115	0.0059	0.0229	5.55E-05	4.0312	95929
	**SD Control (n=322)**			**SD Exenatide (n=322)**			**Wilcoxon signed rank**		
Parameters	Median	Quantiles (25/75)		Median	Quantiles (25/75)		pVal	zVal	signedrank
BFI	26.3603	23.2859	30.3307	32.3823	27.3363	36.8784	5.87E-42	-13.5720	2823
AUC	0.2820	0.1896	0.4229	0.3859	0.1940	0.5780	9.51E-03	-2.5933	20415
SNR	0.1171	0.0778	0.1803	0.0820	0.0469	0.1337	1.25E-05	4.3688	31571
Frequency	0.0185	0.0160	0.0255	0.0250	0.0180	0.0305	5.35E-14	-7.5230	11834
Prominence	0.0141	0.0090	0.0211	0.0178	0.0093	0.0340	1.23E-01	-1.5443	22096
	**SHR Control (n=227)**			**SHR Exnatide (n=227)**			**Wilcoxon signed rank**		
Parameters	Median	Quantiles (25/75)		Median	Quantiles (25/75)		pVal	zVal	signedrank
BFI	30.6420	23.3091	41.2179	40.3754	36.1344	49.8279	2.34E-30	-11.4503	1558
AUC	0.2178	0.1254	0.3884	0.2604	0.1608	0.4032	2.01E-01	-1.2779	11568
SNR	0.0780	0.0575	0.1113	0.0399	0.0291	0.0685	2.27E-11	6.6873	19406
Frequency	0.0205	0.0170	0.0245	0.0270	0.0200	0.0300	3.44E-10	-6.2777	6363
Prominence	0.0115	0.0059	0.0229	0.0126	0.0073	0.0187	2.56E-01	-1.1356	11708

Table 1. Summary statistics for hemodynamic parameters
